# A sonography assisted technique for the removal of a femoral interlocking nail – a technical note

**DOI:** 10.1186/1471-2474-6-51

**Published:** 2005-10-17

**Authors:** Kai-Jow Tsai, Po-Wen Shen, William C Hutton

**Affiliations:** 1 Department of Orthopaedic Surgery Cathay General Hospital, Taipei, Taiwan; 2 Emory Orthopaedic and Spine Center, Department of Orthopaedic Surgery, Emory University, Atlanta, GA, USA

## Abstract

**Background:**

Open methods for removal of femoral interlocking nails involve an incision (up to 10 cm) over the trochanter to find the tip of the nail. The distal locking screws are some times difficult to palpate and an incision (up to about 5 cm) is often needed for exposure. Intra-operative fluoroscopy is often used as an adjunct technique to minimize the surgical wound. However, patients and surgeons are exposed to a radiation hazard. Sonography can provide a real-time and efficient alternative to fluoroscopy.

**Methods:**

Sonography of soft tissue has been established to identify a foreign body. A metallic implant has a hyperechoic image; therefore, we can identify the correct position of the screws preoperatively and intraoperatively.

**Results:**

We have developed a technique using sonography and minimal incisions for the removal of a femoral interlocking nail. The proximal wound is 2.5 cm in length and the wound is 0.5 cm in length for each distal locking screw.

**Conclusion:**

The sonography can be used to minimize the length of incision and prevent radiation exposure in the removal of intramedullary femoral nails.

## Background

The development of closed interlocking intramedullary nailing has allowed the treatment of femoral diaphyseal fractures to become safer and more effective [1,2]. The nail is usually inserted under fluoroscopic control which brings concern over the radiation exposure [3]. There have been efforts to minimize the fluoroscopic radiation [4]. Ultrasound, on the other hand, is cheaper and more easily available and can be used to monitor alignment during closed femoral nailing [5]. Thus, using ultrasound can reduce the fluoroscopic monitoring time and reduce the radiation exposure to the patient and the surgeon.

It is often necessary to remove femoral nails after bony union. Conventional open methods require up to a 10 cm incision over the trochanter. The distal locking screws are difficult to palpate, and open distal incisions are often needed. Fluoroscopy is frequently used in an attempt to decrease the size of the wound.

Sonography for evaluation of soft tissues has been in use for years. The sonographic signal is reflected by cortical bone [6], and any metallic implant has a hyperechoic image. Therefore sonography can identify the position of locking screws. We applied sonography for the wound of the removal of distal locking screws using a minimal incision. We report on this technique that was used successfully in three patients.

## Methods

### Preoperative localization by sonography

**Figure 1:**
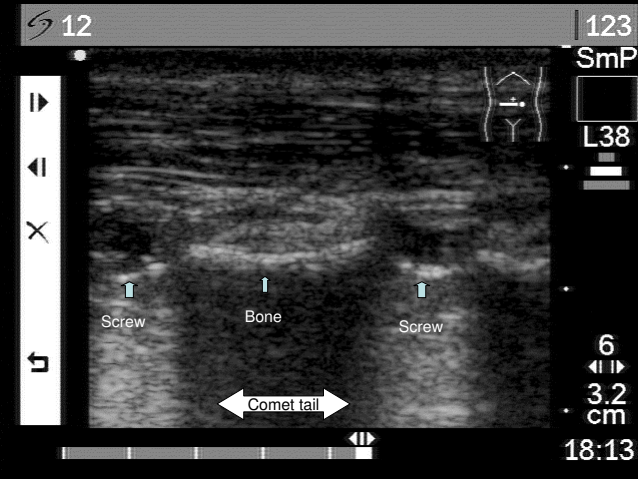
**Sonography of screws**. Two distal locking screws can be visualized by sonography. The image of two extremely brightness with "comet-tails" indicate the distal locking screw heads.

After bony union was achieved, the patient had surgery for the removal of the intramedullary nail. At this time the swelling had subsided and the scar contracture was not aligned to indicate the position of the distal locking screw head. The conventional method to find the screw is to open the skin, fascia, and muscles and expose the bony cortex; the screw is then extracted under direct vision. Using this conventional method there is extensive invasion of the soft tissues. Although by using palpation or intra-operative fluoroscopy, the surgeon can sometimes locate and remove the screws without direct vision. We used Sonosite^® ^180 plus (Bothell Washington) with L38/10.5 MHz linear array for the purpose. The metal implants have a characteristic sonographic appearance. The wide difference in acoustic impedance between soft tissue and metal results in an extremely bright interface, with a posterior "comet-tail" reverberation artefact [7]. The image of the two hyperechoic reflection and comet-tail indicates the distal locking screw heads (Figure 1). A 0.5-cm incision is made over the screw head and the screwdriver is inserted under real-time sonographic assistance.

### Locking screws removal

**Figure 2:**
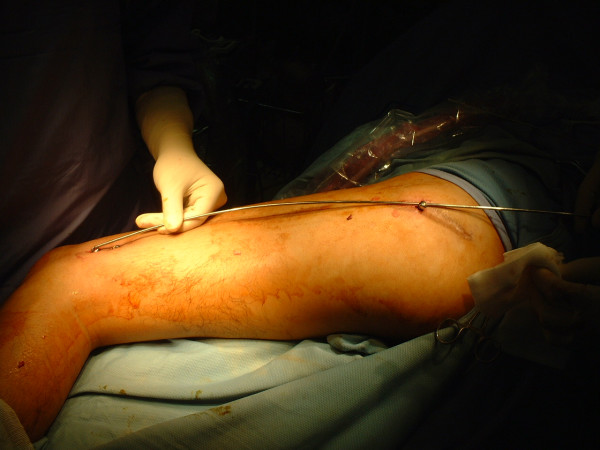
**Long axis of femur**. The three points of the locking screws form a line that indicates the long axis of femur.

There were two distal locking screws and one proximal locking screw. These three screws form the long axis of femoral canal. The long axis is guidance for removal of the nail. The screws are not removed completely but left protruding from the skin to indicate the long axis of femoral shaft (Figure 2).

### The 2.5-cm incision on the tip of the long axis over the buttock

**Figure 3:**
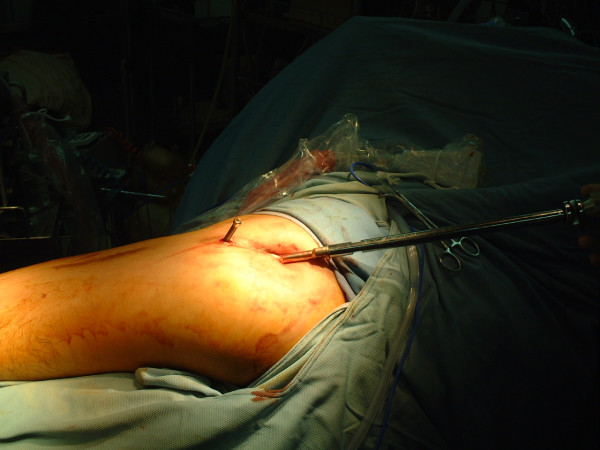
**Tube sleeve**. The custom-made tube sleeve is used in the minimal invasive technique for soft tissue protection.

The skin incision for the removal of the intramedullary nail is at the tip of the long axis over the buttock. The muscles over piriformis fossa were dissected with fingers and the guide pin was inserted through the fascia. The bony structure can be palpated by guide pin. While these three locking screws revealed the long axis of intramedulary nail, the guide pin is inserted to the nail in the medullary cavity. The custom-made tube sleeve is used in the minimally invasive technique for soft tissue protection (Figure 3). The sleeve maintains the direction and prevent any injury to muscles, nerve and vessels [8].

## Results

**Figure 4:**
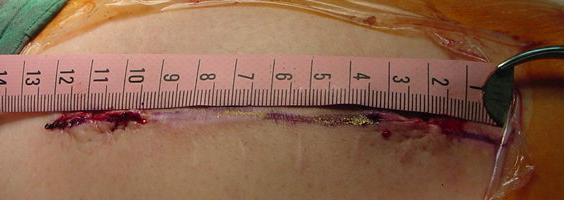
**2.5 cm proximal wound**. The proximal wound is 2.5 cm in length, which is smaller than previous wound that is 10 cm in length.

The authors have used this technique successfully in three patients with no failures. We present a typical patient who suffered from a fall that induced a fracture of his left femur. He was treated with a femoral interlocking nail and the fracture eventually healed one year later. The removal of the interlocking nail was performed using this minimally invasive technique. The proximal wound was about 2.5 cm in length; the previous wound had been about 10 cm in length (Figure 4).

## Discussion

The applications of sonography to removal of surgical implant have been documented in gynecology literature. Nelson et al reported on real-time sonographic localization and guidance could enable safe removal of deeply placed, nonpalable and intramuscular contraceptive capsules [9]. High resolution sonography allowed accurate localization of a foreign body in the soft tissue in spite of radio-lucent or radio-opaque [10].

Gynaecologists are familiar with sonography, while orthopaedic surgeons are familiar with fluoroscopy. Intra-operative fluoroscopy has been widely used for many procedures, such as closed reduction, internal fixation and removal of implant. However, the removal of implant can also be achieved by sonography, because the metal implants located on the surface of bone and have high echogenecity and are well distinguished from the other tissues [11].

The sonography of musculoskeletal system has various applications including detection of an occult fracture, reduction of fracture, assessment of joint fluid, and identification of a foreign body[5,6,12,13]. During the removal of a metal implant, the sonography can provide real-time guidance to apply the screw driver to the screw head and thus assist in the removal of screws.

The authors have used this procedure to remove three femoral interlocking nails. However, most high frequency probes have a limited depth of view, typically 3–4 cm at 12 MHz. When assessing deeper structures, 5-MHz curvilinear probes can give a deeper and wider view [7]. Therefore, the morbidity obesity, heterotopic bone growth or deep seated implant in bone may be not feasible for this technique. The surgeons should consider another modality such as intra-operative fluoroscopy or conventional open procedures.

## Conclusion

The sonography can be used to minimize the incision length and radiation exposure in the removal of intramedullary femoral nails.

## Competing interests

The author(s) declare that they have no competing interests.

## Authors' contributions

KJT: Original idea for this procedure, organize and write the text.

PWS: Advice and support.

WCH: Advice and write the text.

## Pre-publication history

The pre-publication history for this paper can be accessed here:


